# Iron-dependent CDK1 activity promotes lung carcinogenesis via activation of the GP130/STAT3 signaling pathway

**DOI:** 10.1038/s41419-019-1528-y

**Published:** 2019-04-01

**Authors:** Yanbin Kuang, Wenzheng Guo, Jing Ling, Dongliang Xu, Yueling Liao, Hui Zhao, Xiaohui Du, Han Wang, Mingxin Xu, Hongyong Song, Tong Wang, Bo Jing, Kaimi Li, Min Hu, Wenjuan Wu, Jiong Deng, Qi Wang

**Affiliations:** 1 Department of Respiratory Medicine, The Second Affiliated Hospital, Dalian Medical University, Dalian, China; 2 Key Laboratory of Cell Differentiation and Apoptosis of Chinese Minister of Education, Shanghai, China; 3 Department of Laboratory Medicine, Shanghai East Hospital, Tongji University School of Medicine, Shanghai, China; 4 Department of Oncology, Shanghai General Hospital, Shanghai Jiao Tong University School of Medicine, Shanghai, China; 5 Department of Health Examination Center, The Second Affiliated Hospital, Dalian Medical University, Dalian, China; 6 Department of Scientific Research Center, The Second Affiliated Hospital, Dalian Medical University, Dalian, China; 7 Department of Pharmacy, The Second Affiliated Hospital, Dalian Medical University, Dalian, China

## Abstract

Iron dysregulation is associated with several diseases, including lung cancer, but the underlying mechanism is yet unknown. Iron directly binds CDK1, which is upregulated in several cancers, thereby promoting JAK1 phosphorylation and activation of STAT3 signaling to promote colorectal carcinogenesis. This study aimed to investigate the role of iron/CDK1/STAT3 signaling in lung carcinogenesis. We found that iron-dependent CDK1 activity upregulated IL-6 receptor subunit GP130 post-transcriptionally via phosphorylation of 4E-BP1, which is critical for activation of JAK/STAT3 signaling. CDK1 and STAT3 are essential for iron-mediated colony formation in lung cancer cell lines. CDK1 knockdown and iron chelator DFO decreased tumorigenicity and GP130/STAT3 signaling in vivo. Moreover, CDK1/GP130/STAT3 signaling were elevated in lung cancer tissues compared with adjacent normal lung tissues. Altogether, the present results suggest that CDK1 inhibition and iron deprivation are potential strategies to target GP130/STAT3 signaling to suppress lung cancer.

## Introduction

Lung cancer is the second most common cancer and the leading cause of cancer-related mortality among men and women worldwide^1^; however, its underlying mechanism remains unclear. Hence, a better understanding of the molecular mechanism underlying lung carcinogenesis would contribute to the development of novel strategies for its prevention and targeted therapy.

Cyclin-dependent kinases (CDKs) are critical drivers of cell cycle transition. In particular, CDK1 is a key determinant of mitotic progression, and its activity is dysregulated via indirect genetic alteration in tumorigenesis^2^. Integration of gene expression data from different databases (TCGA and GEO) demonstrated CDK1 upregulation in lung adenocarcinoma. Furthermore, CDK1 upregulation is associated with a poor prognosis^3^. Selective targeting of CDK1 might constitute a novel strategy for tumor treatment in certain contexts. CDK1 inhibition is selectively lethal in MYC-dependent human breast cancer cells^4^. As CDK1 activity is essential for the formation of BRCA-1 foci, its inhibition, combined with PARP inhibition, could effectively reduce BRCA-proficient tumor growth and regression^5^. CDK1 inhibition has reportedly increased the efficacy of sorafenib in hepatocellular carcinoma^6^. However, the molecular mechanisms and potential applications of CDK1 in lung cancer remain undetermined.

Iron is an essential nutrient, and its excess or deficient levels may affect diverse biological processes^7^. Altered iron metabolism is closely associated with cancer initiation and progression. Epidemiological studies have reported that high-dietary iron intake significantly elevate the risk of several malignancies, including esophageal cancer, colorectal cancer, liver cancer, and lung cancer^8^. Consistent with epidemiological data, experimental studies have reported that a high-iron diet promotes spontaneous colorectal tumorigenesis and mammary tumor growth in mice^9,10^. On the contrary, a low-iron diet reportedly decreased tumor growth in mice^11^. Iron chelators inhibit human lung tumor xenografts^12^. In addition, a low-iron diet or the use of iron chelators protected cigarette-smoke-induced chronic obstructive pulmonary disease (COPD)^13^, which is related to 4.5-fold higher incidence of lung cancer than in the general population^14^. Altogether, these studies suggested the important role of iron in lung carcinogenesis; however, the underlying molecular mechanism remains unknown. Recently, iron was reported to bind CDK1 directly and enhance its activity. CDK1 phosphorylates JAK1 to activate the JAK/STAT3 signaling pathway and promote colorectal tumorigenesis^9^. However, in the presence of CDK1, a combination of IL-6 and iron elevated STAT3 activity to a greater extent than iron alone^9^. This implies the existence of the yet unrevealed mechanism underlying iron-dependent CDK1-mediated STAT3 activation, such as the regulation of IL-6 receptor subunit GP130 by iron-dependent CDK1 activity.

GP130 is the central player of the receptor complexes formed by IL-6 cytokines. When IL-6 binds IL-6Rα, GP130 dimerizes and activates JAK/STAT3 signaling^15^. IL-6 and related cytokines are critical lynchpins between inflammation and cancer^16^. Systemic and pulmonary IL-6 levels are reportedly elevated in patients with lung adenocarcinoma and these elevations are associated with poor patient survival^17–20^. IL-6 promotes KRAS-driven lung carcinogenesis, and genetic ablation of either IL-6 or STAT3 suppresses the extent of lung cancer^21^. Moreover, blockade of IL-6/STAT3 axis reportedly suppressed cachexia in the same model^22^. These evidences above suggest that regulation of GP130 might suppress lung cancer.

In this study, we investigated the association among iron, CDK1, and GP130/STAT3 signaling in lung carcinogenesis. Our results may contribute to the development of potential strategies to target GP130/STAT3 signaling and suppress lung cancer via CDK1 blockade and iron deprivation.

## Results

### CDK1 and STAT3 are essential for iron-mediated colony formation in lung cancer cell lines

**Fig. 1:**
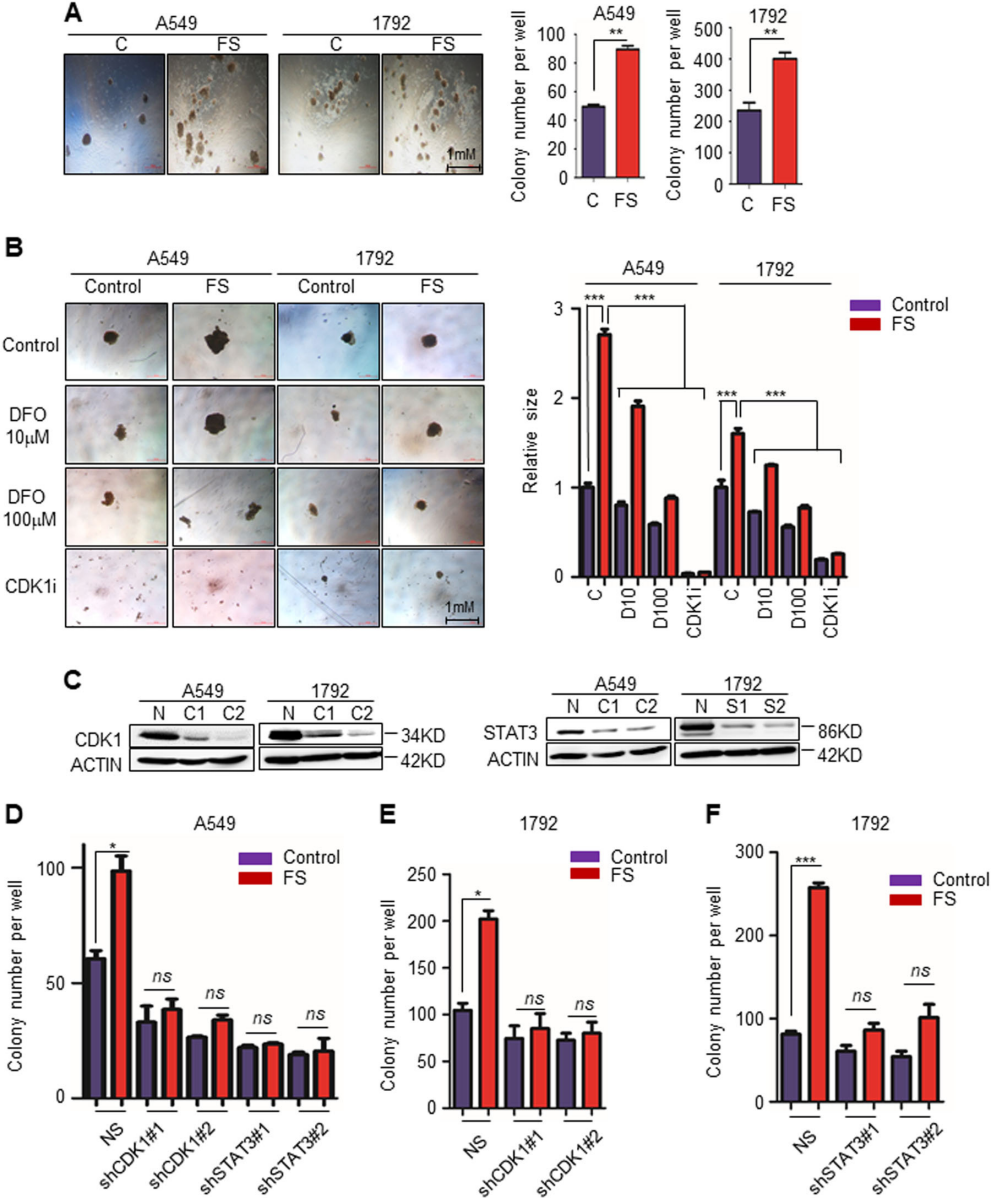
CDK1 and STAT3 are essential for iron-mediated colony formation in lung cancer cell lines. **a** Representative bright-field images and quantification of colonies of A549 or 1792 cells per well in RPMI 1640 medium supplemented with 0 (C) or 100 μM ferrous sulfate (FS) in a soft-agar colony formation assay. **b** Representative bright-field images and quantification of the sphere size in sphere culture assay. A549 or 1792 cells cultured in RPMI 1640 supplemented with 0 (Control) or 100 μM ferrous sulfate (FS), treated with dimethyl sulfoxide (Control), iron chelator DFO (10 μM or 100 μM), and CDK1-selective inhibitor RO-3306 (CDK1i; 10 μM). **c** Western blot analysis for CDK1 or STAT3 expression in A549 and 1792 expressing shRNA against CDK1 (C1 and C2), STAT3 (S1 and S2) or non-specific shRNA NS(N). **d**–**f** Quantification of colonies per well of the indicated cell lines in RPMI 1640 medium supplemented with 0 (Control) or 100 μM ferrous sulfate (FS). *p < 0.05, **p < 0.01, ***p < 0.001. Error bars represent the SEM values

To investigate whether iron promotes lung carcinogenesis, we first performed a soft-agar colony formation assay and a sphere culture assay using lung adenocarcinoma cell lines A549 and 1792. As expected, addition of ferrous sulfate (FS,100 μM) (Sigma-Aldrich) in RPMI 1640 culture medium significantly increased the number of colonies in both lung cancer cell lines (Fig. 1a). We further seeded lung cancer cells into low-adherent 96-well plates, cultured them with different conditional media for 12 days, and then observed the size of the spheres. Larger spheres were observed in the FS (100 μM) group, which was reversed upon addition of an iron chelator deferoxamine (DFO; 100 μM). Even a low DFO concentration (10 μM) suppressed sphere formation in the control group (not treated with FS in the culture medium) (Fig. 1b).

Iron reportedly binds CDK1 directly and enhances its kinase activity. Iron-dependent CDK1 activity promotes colorectal carcinogenesis via the JAK/STAT3 signaling pathway^9^. To investigate whether CDK1 and STAT3 are essential for iron-mediated colony formation in lung cancer cell lines, we cultured lung cancer cells in low-adherent 96-well plates with RPMI 1640 supplemented with 0 or 100 μM FS, and treated the cells with CDK1-selective inhibitor RO-3306 (CDK1i, 10 μM) and STAT3-selective inhibitor Stattic (5 μM) for 12 days. Both CDK1 and STAT3 inhibition significantly decreased sphere formation, regardless of ferrous sulfate addition (Fig. 1b and Supplementary Fig. 1A). We further used shRNA against CDK1 and STAT3, and established corresponding cell lines with a stable knockdown (Fig. 1c). Knockdown of either CDK1 or STAT3 blocked iron-mediated colony formation (Fig. 1d–f).

### Iron-dependent CDK1 activity promotes STAT3 signaling via upregulation of GP130

**Fig. 2:**
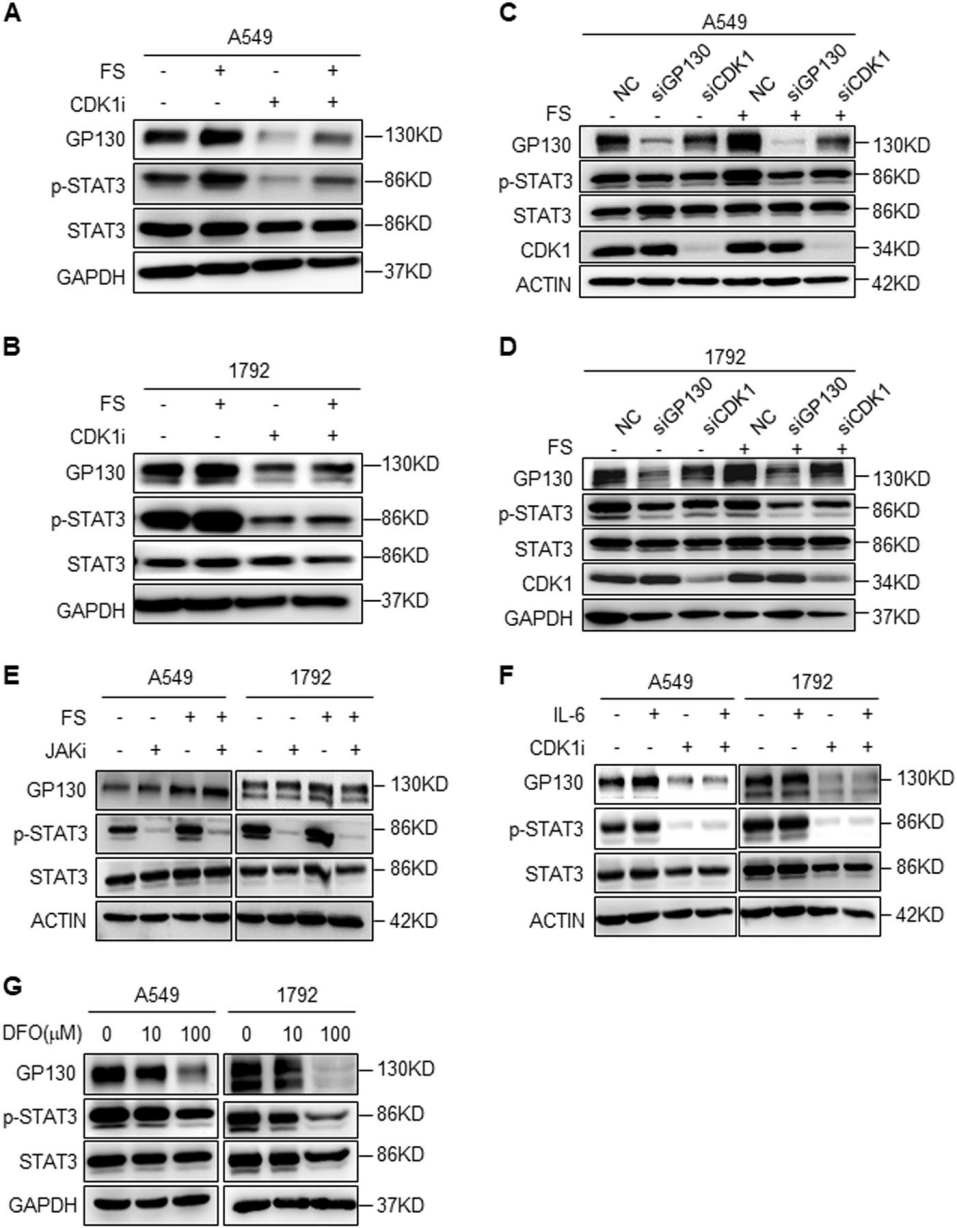
Iron-dependent CDK1 promotes STAT3 signaling via upregulation of GP130 **a**, **b** Western blot analysis for GP130, p-STAT3(Y705), and STAT3 expression in A549 or 1792 cells treated with ferrous sulfate (FS; 100 μM) and CDK1i (10 μM) for 24 h. **c**, **d** Western blot analysis for GP130, p-STAT3(Y705), STAT3, and CDK1 expression in A549 or 1792 cells transfected with siNC (non-specific control)/siGP130/siCDK1, treated with 0 or 100 μM FS. **e** Western blot analysis for GP130, p-STAT3(Y705), and STAT3 expression in A549 or 1792 cells treated with FS (100 μM) and JAK1/2 inhibitor Ruxolitinib (JAKi, 3 μM) for 24 h. JAK inhibition decreased STAT3 signaling; however, it did not affect GP130 expression. **f** Western blot analysis for GP130, p-STAT3(Y705), and STAT3 expression in A549 or 1792 cells treated with IL-6 (10 ng/ml) and CDK1i (10 μM) for 24 h. **g** Western blot analysis for GP130, p-STAT3(Y705), and STAT3 expression in A549 or 1792 cells treated with iron chelator DFO (10 or 100 μM) for 24 h

It was previously reported that CDK1 phosphorylates JAK1 directly and activates downstream STAT3 signaling. However, from previous work, IL-6 seemed to increase the effect on STAT3 activation^9^. We speculate that GP130, an IL-6 receptor and upstream receptor of JAK/STAT3 signaling, might also be regulated by iron-dependent CDK1 activity. To verify our speculation, we performed western blot analysis to assess GP130 expression and STAT3 activation under different conditions. Iron upregulated GP130 and activated STAT3, whereas CDK1i significantly downregulated GP130 and inactivated STAT3 regardless of FS addition (Fig. 2a, b, Supplementary Fig. 1C, and 1D). Consistent with the data on CDK1i, siRNA-mediated knockdown of CDK1 downregulated GP130 and iron-mediated STAT3 activation (Fig. 2c, d). To further verify GP130-mediated STAT3 activation, we knocked down GP130 and observed a reduction in STAT3 activation regardless of FS addition (Fig. 2c, d). JAK1/2 inhibitor Ruxolitinib (JAKi, 3 μM) robustly blocked JAK/STAT3 signaling; however, it did not affect GP130 expression, indicating that GP130 upregulation was not induced by JAK/STAT3 downstream signaling (Fig. 2e). We further treated lung cancer cells with IL-6 (10 ng/ml) in the presence of CDK1i or not, and found CDK1i downregulated GP130 and blocked STAT3 phosphorylation as well (Fig. 2f).

Furthermore, we treated lung cancer cells with different concentrations of iron chelator DFO (10 μM or 100 μM). As expected, GP130 was downregulated and STAT3 phosphorylation was decreased upon iron chelation at different degrees (Fig. 2g). Iron increased reactive oxygen species (ROS) levels via the Fenton reaction and oxidative stress triggered STAT3 signaling^23,24^. To exclude the possibility that oxidative stress activates STAT3 signaling and promotes sphere formation directly in our study, we treated lung cancer cells with the antioxidant butylated hydroxyanisole (BHA, 100 μM). However, it failed to suppress sphere formation and iron-mediated GP130/STAT3 signaling (Supplementary Fig. 1A, B).

### Iron-dependent CDK1 activity post-transcriptionally upregulates GP130

**Fig. 3:**
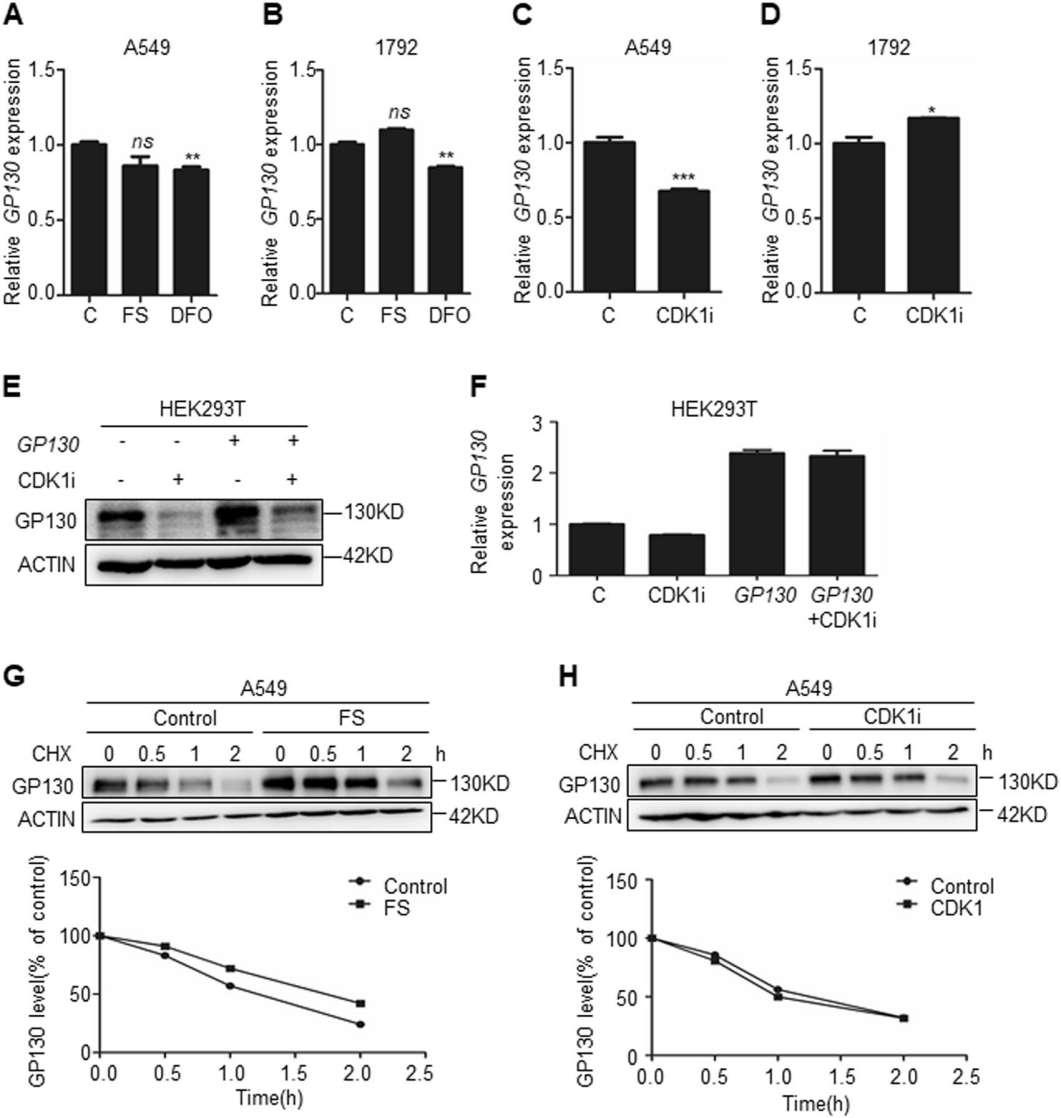
Iron-dependent CDK1 post-transcriptionally upregulates GP130. **a–d** The relative GP130 mRNA expression quantified via quantitative polymerase chain reaction (qPCR) for the indicated cell lines treated with ferrous sulfate (FS; 100 μM) or iron chelator DFO (100 μM) or CDK1i (10 μM) or untreated control (C) for 24 h. (*n* = 3). **e** Western blot analysis for GP130 expression in the HEK293T cell line transfected with *GP130* or vector control, treated with 0 or 10 μM CDK1i for 24 h. **f** Relative GP130 mRNA expression quantified via qPCR in HEK293T cell line transfected with *GP130* or vector control, treated with 0 or 10 μM CDK1i for 24 h. (*n* = 3). **g**, **h** Western blot analysis for GP130 expression in A549 cells and the relative amounts of GP130 were calculated after normalization (GP130/ACTIN). At the beginning of each chase, cycloheximide (CHX, 30 μg/ml) was added to block translation, along with CDK1i (0 or 10 μM). FS (0 or 100 μM) pre-treatment was administered for 24 h. Cells were harvested at the indicated time point. *p < 0.05, **p < 0.01, ***p < 0.001. Error bars represent the SEM values

To understand how iron-mediated CDK1 upregulates GP130, we initially quantified *GP130* mRNA levels via quantitative real-time polymerase chain reaction (qPCR) under different conditions. *GP130* mRNA levels did not increase significantly upon treatment with FS. GP130 mRNA showed in both A549 and 1792 a slight but statistically significant decrease upon treatment with the iron chelator DFO (Fig. 3a, b), suggesting that treatment with this agent might affect to a small extent the transcriptional regulation of GP130. CDK1i partially downregulated GP130 mRNA in A549 cells (Fig. 3c), but it upregulated GP130 mRNA in 1792 cells (Fig. 3d), suggesting that the possible contribution of CDK1 to GP130 mRNA regulation could differ depending on the context. To determine whether CDK1 affects GP130 protein level via regulation of GP130 transcription, we overexpressed GP130 in the HEK239T cell line via transfection of the *GP130* plasmid, which maintained upregulated *GP130* mRNA. Thereafter, these cells were treated with CDK1i for 24 h, and GP130 protein was significantly downregulated regardless of *GP130* mRNA expression (Fig. 3e, f).

We further analyzed whether GP130 protein stabilization was regulated by iron-dependent CDK1 activity. At the beginning of each chase, cycloheximide (CHX, 30 μg/ml) was added to block translation. CDK1i (10 μM) was added at the beginning of each chase. FS (100 μM) pre-treatment was administered for 24 h. Cells were harvested at the indicated time point. Iron or CDK1i did not affect the rate of GP130 degradation a lot (Fig. 3g, h). We further added MG132 and CHL to inhibit proteasome-dependent and lysosome-dependent degradation; however, these inhibitors did not reverse CDK1i- or siCDK1-mediated GP130 downregulation (Supplementary Fig. 2A–D). Altogether, these findings suggest that iron-dependent CDK1 activity upregulates GP130 post-transcriptionally.

### Iron-dependent CDK1 activity promotes GP130 cap-dependent translation via phosphorylation of 4E-BP1

**Fig. 4:**
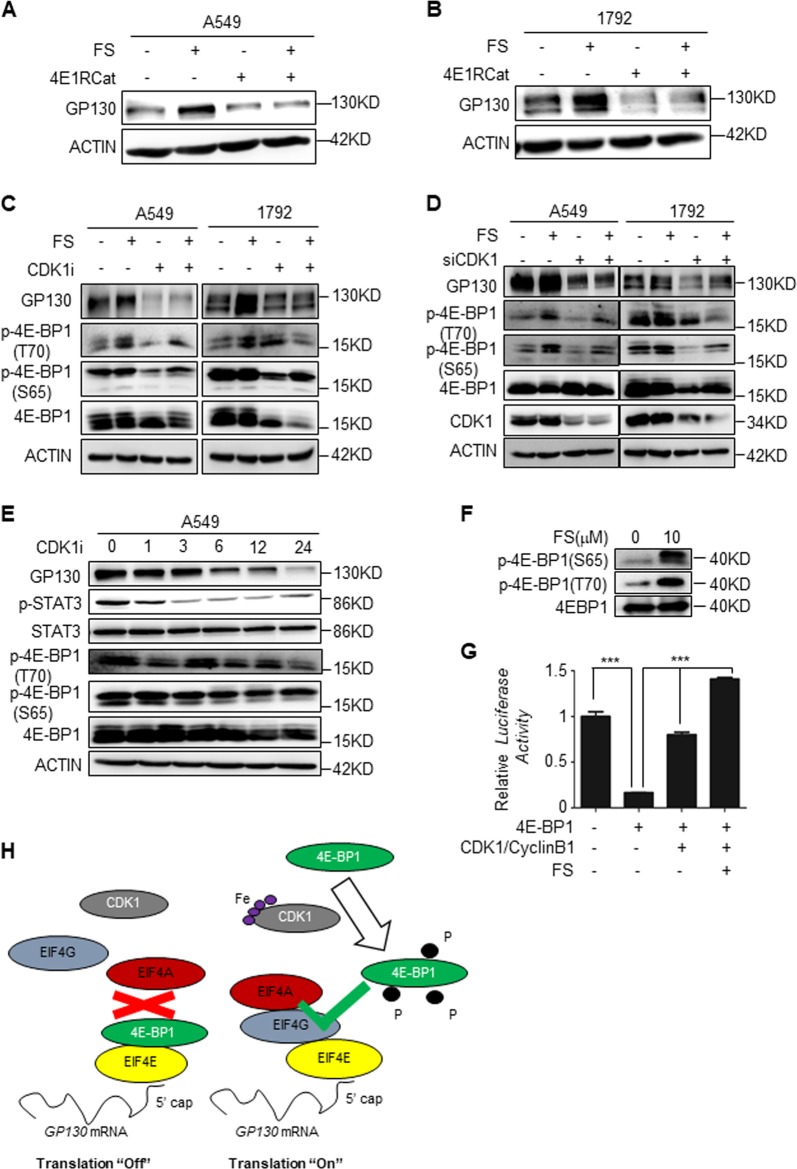
Iron-dependent CDK1 promotes GP130 cap-dependent translation via 4E-BP1 phosphorylation. **a**, **b** Western blot analysis for GP130 expression in A549 or 1792 cells treated with ferrous sulfate (FS; 100 μM) and cap-dependent translation inhibitor 4E1RCat (50 μM) for 24 h. **c** Western blot analysis for GP130, p-4E-BP1(T70), p-4E-BP1(S65), and 4E-BP1 expression in A549 or 1792 cell lines treated with FS (100 μM) and CDK1i (10 μM) for 24 h. **d** Western blot analysis for GP130, p-4E-BP1(T70), p-4E-BP1(S65), and 4E-BP1 expression in A549 or 1792 transfected with siNC/siCDK1, treated with FS (100 μM) for 24 h. **e** Western blot analysis for GP130, p-STAT3, p-4E-BP1(T70), p-4E-BP1(S65), and 4E-BP1 expression in A549 cells treated with CDK1i (10 μM) for indicated time. **f** In vitro phosphorylation assay to assess 4E-BP1 phosphorylation. Recombinant GST-CDK1/CyclinB1 and GST-4E-BP1 were added into 1x protein kinase buffer in the presence 0 or 10 μM FS, Supplemented with 200 μM ATP, and incubated at 30 °C for 30 min. The reactions were stopped by adding loading buffer and boiled at 95 °C for 5 min. Then these protein samples were subjected to western blot. **g** In vitro protein translation assay. Forty microliters of TNT® Quick Master Mix, 1 μl Methionine (1 mM), 2 μl pcDNA3.1-Luciferase plasmid, and 7 μl in vitro protein phosphorylation reaction products from indicated conditions, were incubated at 30 °C for 90 min. At last, we evaluated the luciferase translation level. **h** Schematic representation of hypophosphorylated 4E-BP1 sequestering eIF4E from the eIF4F cap initiation complex and turning “Off” GP130 cap-dependent translation. However, when iron binds CDK1 and enhances its activity, 4E-BP1 is hyperphosphorylated and cap-dependent translation is turned “On”

CDK1 reportedly activates cap-dependent translation by directly hyperphosphorylating 4E-BP1^25,26^. Hypophosphorylated 4E-BP1 could sequester eIF4E from the eIF4F cap initiation complex, but when 4E-BP1 is hyperphosphorylated, it lost this ability to bind eIF4E. Furthermore, hypophosphorylated 4E-BP1 is degraded, while hyperphosphorylated 4E-BP1 is refractory to degradation^27^. Previous studies classified 4E-BP1 into four groups according to its molecular weight and named α, β, γ, and δ. CDK1 phosphorylates 4E-BP1 at T37, T46, S65, T70, and S83 site, resulting in a mitosis-specific hyperphosphorylated δ isoform^25,26^. However, we notice that mutation of 4E-BP1 at S83 site didnot affect general cap-dependent translation initiation complex formation^26^. This result might suggest that the key point of translation initiation complex formation is more likely 4E-BP1 hyperphosphorylation than 4E-BP1 phosphorylated at specific sites. Therefore, we speculate that iron-dependent CDK1 activity upregulates GP130 via a 4E-BP1 hyperphosphorylation mechanism. To confirm whether GP130 protein synthesis is cap-dependent, we used cap-dependent translation inhibitor 4E1RCat, which prevents eIF4G from binding eIF4E, similar to the role of 4E-BP1. 4E1RCat markedly downregulated GP130 regardless of addition of iron (Fig. 4a, b). Thereafter, we investigated whether iron and CDK1 are correlated with 4E-BP1 phosphorylation. A549 and 1792 cells were treated with FS (100 μM) and CDK1i (10 μM) for 24 h, and iron promoted 4E-BP1 phosphorylation at Ser65/Thr70, which was reversed by CDK1i (Fig. 4c). Consistent with the data on CDK1i, siRNA-mediated knockdown of CDK1 decreased GP130 expression and inhibited 4E-BP1 phosphorylation (Fig. 4d). We treated lung A549 cells with CDK1i for different time and found decreased GP130, p-STAT3, and p-4E-BP1 expression along with time (Fig. 4e). To further confirm our speculation, we performed an in vitro phosphorylation assay, and we found that iron promoted 4E-BP1 phosphorylation via CDK1 (Fig. 4f). Furthermore, in vitro protein translation assay was performed with TnT® Quick Coupled Transcription/Translation System (L1170; Promega). 4E-BP1 inhibited protein translation, but CDK1/CyclinB1 in the presence of 0 or 10 μM FS phosphorylate 4E-BP1 and increase protein translation at different degrees (Fig. 4f, g). Therefore, we proposed that hypophosphorylated 4E-BP1 sequesters eIF4E from the eIF4F cap initiation complex and turns GP130 cap-dependent translation “Off.” However, when iron binds CDK1 and enhances its activity, is hyperphosphorylates 4E-BP1 and turns cap-dependent translation “On” (Fig. 4h).

### CDK1 knockdown and iron chelator DFO decrease tumorigenicity and GP130/STAT3 signaling in vivo

**Fig. 5:**
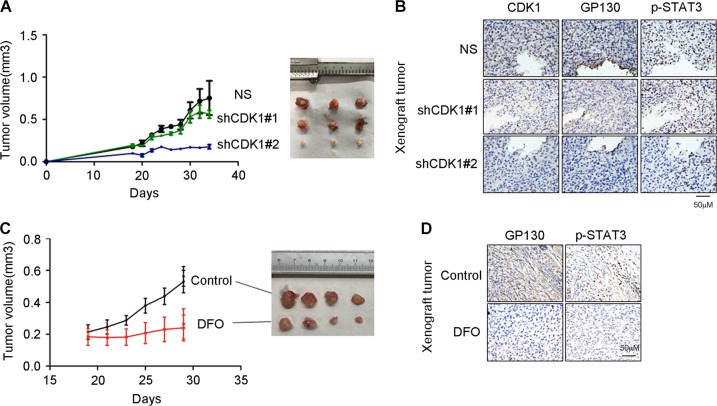
CDK1 knockdown and iron chelator DFO decrease tumorigenicity and GP130/STAT3 signaling in vivo. **a** Nude mice were injected with A549-NS/shCDK1(#1 and #2) cells (1 × 10^6^ cells). Tumor volumes were assessed in the indicated cell lines at the indicated time points. **b** Representative images of immunohistochemical (IHC) staining for CDK1, GP130, and p-STAT3 in A549-NS/shCDK1(#1 and #2) xenograft tumor tissues. **c** Nude mice were injected with A549 cells (1.5 × 10^6^ cells). After 19 days, the mice were administered an intraperitoneal injection of DFO (16 mg/kg) dissolved in 0.9% NaCl (DFO), or saline solution alone (Control), once a day for 12 days. Tumor volumes were assessed at the indicated time points. **d** Representative images of immunohistochemical (IHC) staining for GP130, and p-STAT3 in xenograft tumor tissues of control group (Control) or DFO group (DFO)

To verify whether CDK1 is essential for tumorigenicity in vivo, we established A549-NS, A549-shCDK1#1 and A549-shCDK1#2 cell lines using shRNA against CDK1 or NS (non-specific shRNA sequence). These cell lines were injected subcutaneously into nude mice. The tumorigenicity of A549-shCDK1#2 was significantly lower than that of A549-NS, while that of A549-shCDK1#1 was slightly but not significantly lower than that of A549-NS (Fig. 5a). This may have resulted from minor differences in CDK1 expression between A549-NS and A549-shCDK1#1 (Fig. 5b). Concurrent with our in vitro data, immunohistochemistry staining of xenograft tissue slides indicated GP130 and p-STAT3 downregulation upon CDK1 knockdown (Fig. 5b).

Furthermore, nude mice were injected with A549 cells (1.5 × 10^6^ cells). After 19 days, the mice were administered an intraperitoneal injection of DFO (16 mg/kg) dissolved in 0.9% NaCl, or saline solution alone, once a day for 12 days. DFO significantly decreased tumorigenicity and the expression of GP130 and p-STAT3 (Fig. 5c, d).

### CDK1/GP130/STAT3 signaling were greater in lung cancer tissues than in adjacent normal lung tissues

**Fig. 6:**
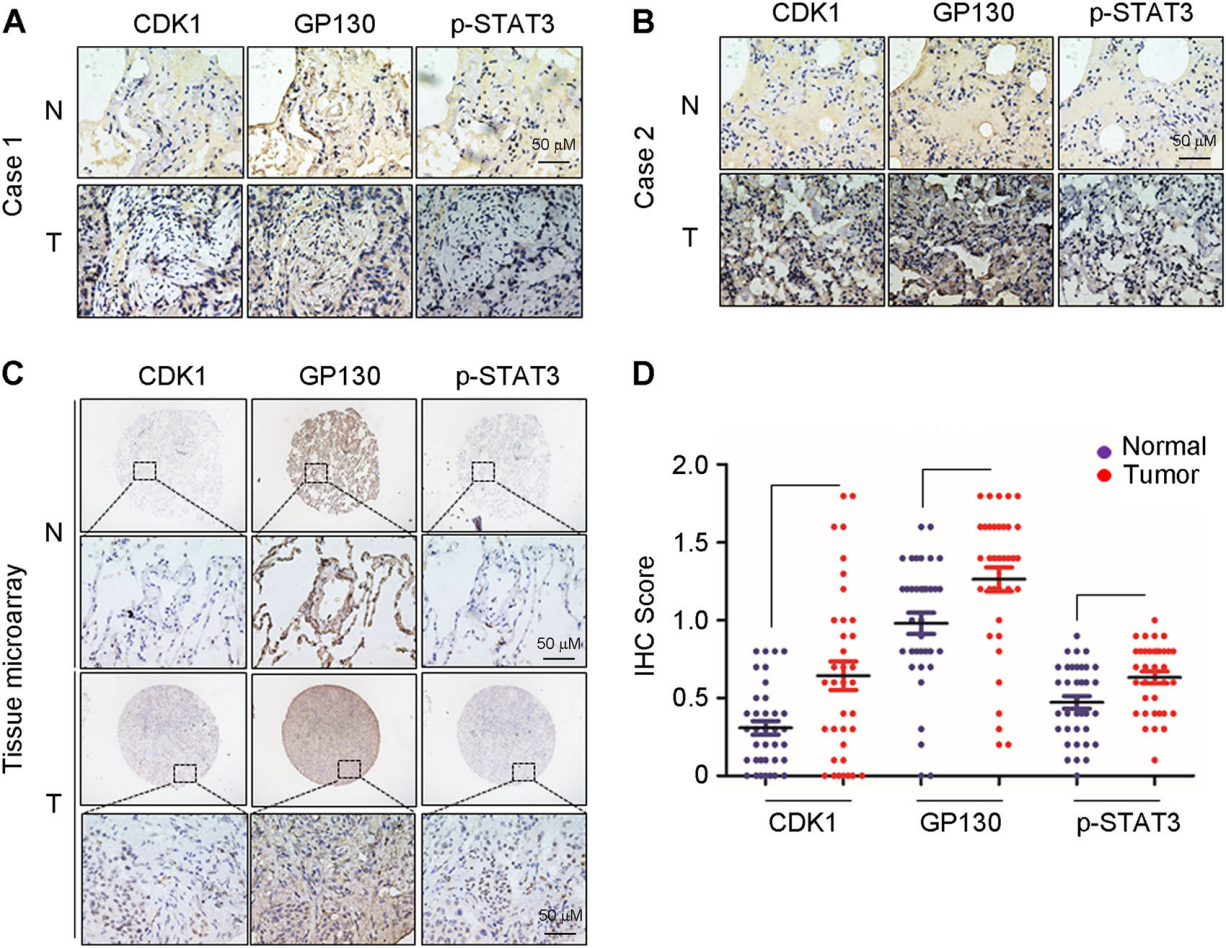
CDK1/GP130/STAT3 signaling were elevated in lung cancer tissues compared with adjacent normal lung tissues. **a**, **b** Representative images of immunohistochemical (IHC) staining for CDK1, GP130, and p-STAT3 in lung cancer tissue (T) and adjacent normal lung tissues (N) from lung cancer patients (Case 1 and Case 2). **c** Representative images of IHC staining for CDK1, GP130, and p-STAT3 in lung cancer tissue (T) and adjacent normal lung tissues (N) from tissue microarrays. **d** IHC staining scores for CDK1, GP130, and p-STAT3 from tissue microarrays. **p < 0.01. Error bars represent the SEM values

To further investigate the clinical relevance of our findings, we performed immunohistochemical staining to assess the CDK1, GP130, and p-STAT3 expression in lung cancer tissues and adjacent normal lung tissues. CDK1, GP130, and p-STAT3 were upregulated in lung cancer tissues compared with adjacent normal lung tissues (Fig. 6a, b). We further stained tissue microarrays comprising 36 pairs of lung cancer tissues and adjacent normal lung tissues. CDK1, GP130, and p-STAT3 were upregulated in lung cancer tissues compared with adjacent normal lung tissues (Fig. 6c). Altogether, these results suggest that elevated CDK1/GP130/STAT3 signaling may essentially contribute to lung carcinogenesis.

## Discussion

In this study, we first proposed that iron-dependent CDK1 activity upregulates GP130 and activates downstream STAT3 signaling. CDK1 and STAT3 are essential for iron-mediated colony formation. CDK1 knockdown and iron chelator DFO decrease tumorigenicity and GP130/STAT3 signaling in vivo. Moreover, CDK1/GP130/STAT3 signaling were elevated in lung cancer tissues compared with adjacent normal lung tissues.

CDK1 is a key determinant of mitotic progression, and its activity is dysregulated by direct genetic alterations during tumorigenesis^2^. CDK1 is upregulated in lung adenocarcinoma and is suggested to have a poor prognosis^3^. Emerging studies suggest that selective targeting of CDK1 might constitute a novel plausible strategy for tumor treatment in certain contexts^4–6^. However, the molecular mechanism and the potential applications of CDK1 with respect to lung cancer are yet unclear. CDK1 promotes cap-dependent translation via 4E-BP1 phosphorylation and might contribute to cell transformation^25,26^. This study is the first, to our knowledge, to propose that iron promotes GP130 cap-dependent translation via CDK1-dependent 4E-BP1 phosphorylation. GP130 is an indispensable receptor of IL-6-type cytokines^15^, and iron deprivation- or CDK1 inhibition-mediated GP130 downregulation markedly blocked STAT3 signaling. IL-6-type cytokines are the critical lynchpins between inflammation and cancer^16^. Systemic and pulmonary IL-6 are elevated in lung adenocarcinoma patients and are related to poor patient survival^17–20^. IL-6 promotes KRAS-driven lung carcinogenesis, and genetic ablation of either IL-6 or STAT3 suppresses the extent of lung cancer^21^. In addition, lung cancer chemosensitivity is reportedly associated with STAT3 signaling^28,29^. Altogether, the present results suggest a potential preventive and treatment strategy for lung cancer via suppression of GP130/STAT3 signaling via CDK1 inhibition or iron deprivation.

Although the present results support the premise that STAT3 signaling promotes lung carcinogenesis, STAT3 reportedly unexpectedly opposed tumorigenesis in KRAS-mutant lung adenocarcinoma, lung epithelial-specific inactivation of Stat3 promoted Kras^G12D^-driven lung adenocarcinoma^30^. The tumor-suppressive role of STAT3 is attributed to its ability to inhibit NF-κΒ-induced transcription of the proangiogenic chemokine *Cxcl1*, thereby suppressing tumor vascularization and tumor progression^30^. Furthermore, STAT3 promotes lung carcinogenesis via IL-6 trans-signaling without altering *Cxcl1* transcription; hence, these results were considered to probably be caused by different STAT3 upstream cytokines, or that STAT3 heterodimers modulated a different network of gene targets^21^. In the present study, iron deprivation or CDK1 inhibition downregulated IL-6-type cytokine receptor GP130, which theoretically blocked both binding of STAT3 upstream cytokines and downstream STAT3 heterodimers signaling.

Epidemiological and experimental data suggest that iron contributes to lung carcinogenesis^8–14^; however, the underlying mechanism is still unknown. Iron reportedly binds CDK1 directly and enhances its activity, CDK1 phosphorylates JAK1 directly, and the activation of JAK/STAT3 signaling promotes colorectal cancer^9^. In the present study, CDK1 upregulated GP130, which then served as the upstream receptor of JAK. This seemingly complements the mechanism underlying CDK1-mediated activation of STAT3 signaling. It was first proposed that iron-dependent CDK1 activity activates GP130/STAT3 signaling to promote lung carcinogenesis. As an important trace element, iron is required for protein and enzyme activity and is closely associated with ferroptosis, a form of cell death caused by the iron-mediated lipid peroxide generation^8^. However, numerous questions regarding the mechanism of action and application of iron in lung cancer remain unanswered.

**Fig. 7:**
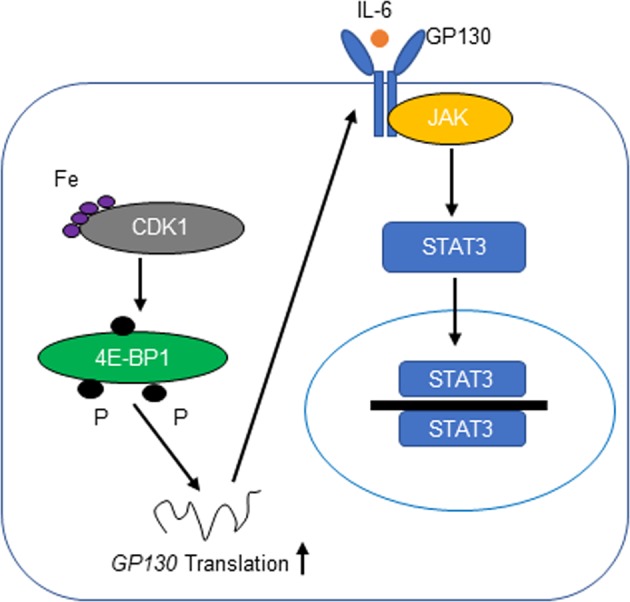
Schematic representation of the mechanism underlying the role of iron in JAK/STAT3 signaling in lung cancer tissue. Iron binds CDK1 and enhances its activity to phosphorylate 4E-BP1, which in turn increases GP130 cap-dependent translation and downstream JAK/STAT3 signaling upon IL-6 binding

In conclusion, the present results indicate that iron binds CDK1, thereby enhancing 4E-BP1 phosphorylation, subsequently increasing GP130 cap-dependent translation and downstream JAK/STAT3 signaling upon IL-6 binding (Fig. 7). The present results further the current understanding of the molecular mechanism underlying the role of iron and CDK1 in lung carcinogenesis.

## Materials and methods

### Cell lines and cell culture

Human lung adenocarcinoma cell lines A549 and 1792 were obtained from ATCC, and cultured in RPMI 1640 medium supplemented with 10% fetal bovine serum. Human embryonic kidney cells HEK293T from ATCC were cultured in DMEM medium supplemented with 10% fetal bovine serum. These cells were characterized/authenticated by DNA typing at Shanghai Jiao Tong University Analysis Core.

### Transfection and stable cell lines generation

GP130 and CDK1 siRNA were purchased from Biomics Biotech. CDK1 and STAT3 shRNA were obtained from Shanghai Jiao Tong University cDNA library. Non-specific shRNA (NS) was used as control. HEK293T cells were transfected with lenti-shRNA, and the lentiviruses-containing media were harvested after 48 h and used to infect A549 and 1792. All transfections were performed with Lipofectamine 2000 (Invitrogen) according to the manufacturer’s protocols. Stably transfected cells were selected with puromycin (2 μg/ml).

### Soft-agar colony formation assay

Melted 2% agar solution combined with indicated conditional medium (RPMI 1640 supplemented with 0 μM or 100 μM FS) at a ratio of 1:3 (v/v) and added into 24-well plates. Plates were placed at 4 °C in refrigerator for 10 min to solidify. Thereafter, Cell suspensions (500/50 μl) were pipetted onto the solidified agar. Top agar layer was added over the cell suspensions, consisting of the 2% agar solution combined with indicated conditional medium (1:6 v/v) along with matrigel (1:30 v/v). The established colonies number were counted after 2 weeks.

### Sphere culture assay

A549 and 1792 cells were seeded into low-adherent 96-well plates (2000 per well), and were cultured in indicated conditional medium. The culture media were replaced every 3 days. After 12 days, the spheres were observed and photographed.

### Western blot

Cells were washed twice by PBS (Hyclone), and then lysed with RIPA buffer (150 mM NaCl, 50 mM Tris, 25 mM NaF, 2 mM Na3VO4, 0.5% deoxycholate,1% NP40) supplement with protease inhibitor cocktail (biomark.cn). Equal amount of protein samples was mixed with loading buffer and boiled at 95 °C for 5 min. Then proteins were separated by SDS-PAGE and transferred to nitrocellulose (NC) membranes. Five percent non-fat milk were used to block non-specific binding sites of the membranes. After that, primary antibody was added on the membranes and incubated at 4 °C overnight. Membranes were washed three times by TBST, then secondary antibody was incubated at room temperature for 1 h. ECL kit (Millipore) was used for Chemiluminescence^31^. CDK1 (sc-8395, sc-137035) and GP130 (sc-655) primary antibody were purchased from Santa cruz. p-STAT3 (#9145), STAT3 (#9139), 4E-BP1 (#9644), p-4E-BP1 Ser65 (#9451), and p-4E-BP1 Thr70 (#9455) were purchased from Cell signaling technology. HRP-ACTIN (HRP-60008) was purchased from proteintech.

### Protein stability

A549 cells was seeded into 6-well plates. At the beginning of each chase, cycloheximide (CHX, 30 μg/ml) was added to block translation. CDK1i (10 μM) was added at the beginning of each chase. FS (100 μM) pre-treatment was administered for 24 h. Cells were harvested at the indicated time point. Western blot analysis for GP130 expression and the relative amounts of GP130 were calculated after normalization (GP130/ACTIN).

### Quantitative real-time PCR

Total RNA was extracted from cells using the RNA simple Total Kit (TIANGEN). cDNA was synthesized using FastQuant RT kit(TIANGEN). Furthermore, Quantitative real-time PCR experiments were using SuperReal PreMix Plus SYBR Green kit (TIANGEN).^31^GP130 mRNA levels were measured using the primer (forward: 5′-CGGACAGCTTGAACAGAATGT-3′ and reverse: 5′-ACCATCCCACTCACACCTCA-3′), GAPDH (forward: 5′-TGCACCACCAACTGCTTAGC-3′ and reverse: 5′-GGCATGGACTGTGGTCATGAG-3′).

### In vitro protein phosphorylation assay and in vitro protein translation assay

One-hundred nanograms of recombinant GST-CDK1/CyclinB1 (SRP5009, Sigma-Aldrich) and 0.2 μg recombinant GST-4E-BP1 (Ag5056, Proteintech) were added into 24 μl 1x protein kinase buffer (#B6022, New England Biolabs) in the presence or absence of 10 μM FS, Supplemented with 200 μM ATP (P0756, New England Biolabs), and incubated at 30 °C for 30 min. The reactions were stopped by adding loading buffer and boiled at 95 °C for 5 min. Then these protein samples were subjected to western blot. In vitro protein translation assay was performed with TnT® Quick Coupled Transcription/Translation System (L1170; Promega) according to the manufacturer’s protocols. Forty microliters TNT® Quick Master Mix, 1 μl Methionine (1 mM), 2 μl pcDNA3.1-Luciferase plasmid, and 7 μl in vitro protein phosphorylation reaction products from indicated conditions, were incubated at 30 °C for 90 min. At last, we evaluated the luciferase translation level via detecting the light intensity upon addition of luciferin.

### Tumorigenicity in nude mice

Human lung adenocarcinoma cell lines A549 were stably transfected. Then A549-NS/A549-shCDK1 cells (1 × 10^6^) combined with matrigel were injected subcutaneously into 8-week-old nude mice. The tumor volume was measured every 2 or 3 days. Nude mice were injected with A549 cells (1.5 × 10^6^ cells) combined with Matrigel. After 19 days, the mice were administered an intraperitoneal injection of DFO (16 mg/kg) dissolved in 0.9% NaCl, or saline solution alone, once a day for 12 days. Tumor volumes were measured every 2 days. All mice were maintained according to a protocol approved by Shanghai Jiao Tong University, School of Medicine Animal Care and Use Committee [experimental animal use permission No: SYXK (Shanghai) 2008–0050] in the specific pathogen-free animal facility in the university.

### Immunohistochemistry (IHC)

NSCLC patients tissue samples obtained from the Second Affiliated Hospital of Dalian Medical University (Dalian, China)^32^. Tissue microarray presented from Jiong Deng’s Lab. Tissue samples were stained to identify CDK1, GP130, and p-STAT3 proteins. The IHC protocol and score method were performed as previously described^33^. All antibodies were diluted for use according to the manufacturer’s instructions.

### Statistical analyses

Data were analyzed using the software GraphPad Prism Version 5.01. Data are presented as the mean ± SD. A two-tailed unpaired *t*-test was used to compare results. *p* < 0.05 was considered statistically significant.

## Supplementary information

Supplementary figure legends

Supplementary Figure 1

Supplementary Figure 2
