# Supplementary material for: Iron-dependent CDK1 activity promotes lung carcinogenesis via activation of the GP130/STAT3 signaling pathway
Source: Cell Death Dis. 2019 Apr 1;10(4):297. doi: 10.1038/s41419-019-1528-y (PMC6443808; doi:10.1038/s41419-019-1528-y)
Supplement: Supplementary file 1 — Supplementary figure legends [file 41419_2019_1528_MOESM1_ESM.docx]

**Supplementary Figure 1.**

(**A**) Representative bright-field images of the sphere culture assay. A549 or 1792 cells cultured in RPMI 1640 supplemented with 0 (Control) or 100 μM ferrous sulfate (FS), treated with dimethyl sulfoxide (Control) or STAT3-selective inhibitor Stattic (5 μM) or antioxidant butylated hydroxyanisole (BHA; 100 μM).

(**B**) Western blot analysis for GP130, p-STAT3(Y705), and STAT3 expression in A549 or 1792 cells treated with FS (100 μM) and BHA (100 μM) for 24 h. BHA failed to downregulate GP130 and STAT3 signaling.

(**C–D**) Western blot analysis for GP130, p-STAT3(Y705), and BCL2 expression in A549 or 1792 cells treated with ferrous sulfate (FS; 100 μM) and CDK1i (10 μM) for 48 h.

**Supplementary Figure 2.**

(**A–B**) Western blot analysis for GP130 expression in A549 or 1792 cells treated with proteasome inhibitor MG132 (20 μM), and lysosome inhibitor CHL (100 μM) for 6 h. All cells were pre-treated with FS (100 μM) and CDK1i (10 μM) for 24 h.

(**C–D**) Western blot analysis for GP130 expression in A549 or 1792 cells transfected with siNC or siCDK1, treated with MG132 (20 μM) and CHL (100 μM) for 6 h. All cells were pre-treated FS (100 μM) for 24 h.
