# Supplementary figures and images for: Iron-dependent CDK1 activity promotes lung carcinogenesis via activation of the GP130/STAT3 signaling pathway
Source: Cell Death Dis. 2019 Apr 1;10(4):297. doi: 10.1038/s41419-019-1528-y (PMC6443808; doi:10.1038/s41419-019-1528-y)

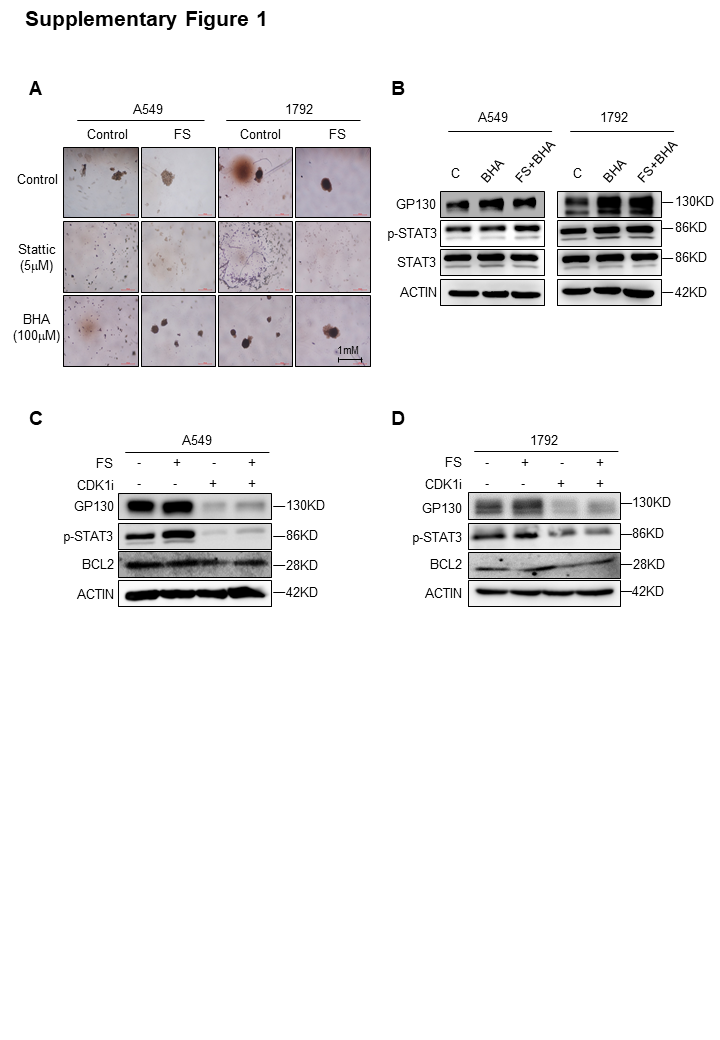

Supplement: Supplementary file 2 — Supplementary Figure 1 [file 41419_2019_1528_MOESM2_ESM.tif]

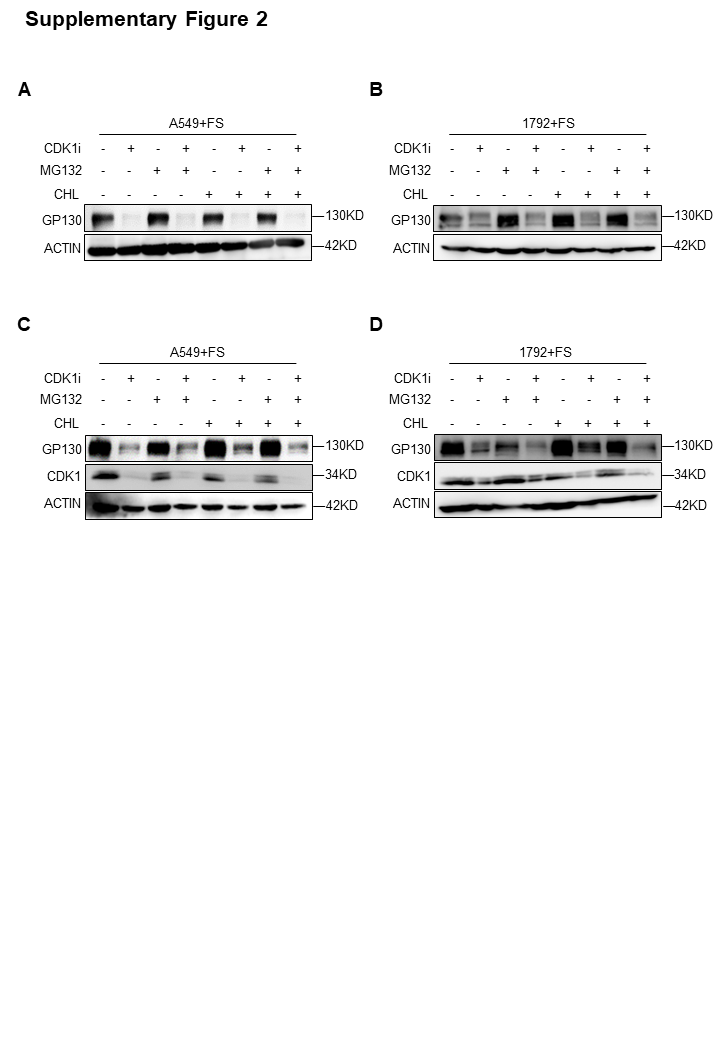

Supplement: Supplementary file 3 — Supplementary Figure 2 [file 41419_2019_1528_MOESM3_ESM.tif]
